# SELENOT, a Key Membrane-Bound Selenoprotein, Mediates Selenium’s Protection Against Lead-Induced Renal Aging in Chickens by Modulating Inflammation and Autophagy

**DOI:** 10.3390/biology15151218

**Published:** 2026-07-23

**Authors:** Yan Wang, Zhiyu Hao, Minna Qiu, Minghang Chang, Xiumei Liu, Yihao Zhu, Hao Wang, Shi Li, Yuhao Liu, Xiaohua Teng, You Tang

**Affiliations:** 1 School of Public Health, Beihua University, Jilin 132013, China; wy@beihua.edu.cn; 2 College of Animal Science and Technology, Northeast Agricultural University, Harbin 150030, China15146551036@163.com (Y.L.); 3 College of Life Sciences, Yantai University, Yantai 264005, China; 4 Heilongjiang Animal Husbandry Service, Harbin 150069, China; 5 Electrical and Information Engineering College, Jilin Agricultural Science and Technology University, Jilin 132101, China

**Keywords:** lead, selenium, membrane-bound selenoproteins, kidney aging, inflammation, autophagy

## Abstract

Lead (Pb) pollution is a global public health issue, yet the mechanisms underlying Pb-induced kidney aging remain poorly understood. Selenium (Se), an essential trace element critical for kidney health, primarily exerts its physiological roles via selenoproteins; among them, membrane-bound selenoproteins strategically localized at the endoplasmic reticulum and plasma membrane, have emerged as potential molecular links in Se-mediated protection. In this study, Hyline chicken models (treated with 350.00 mg/L Pb or/and 1.00 mg/kg Se) and HK-2 cell models (treated with 200 μM Pb or/and 2.5 μM Se) were established to investigate the protective mechanism of Se to Pb poisoning in kidneys, with emphasis on membrane-bound selenoproteins and kidney aging. Results showed that Pb significantly decreased (*p* < 0.05) membrane-bound selenoproteins and induced kidney aging. Inflammation and autophagy were involved, as Pb significantly increased (*p* < 0.05) pro-inflammatory cytokines interleukin-4 (*IL-4*) and interleukin-12β (*IL-12β*), and autophagy-related genes autophagy related 5 (*ATG5*), BCL2 interacting coiled coil protein 1 (*Beclin 1*), and microtubule-associated protein 1A/1B-light chain 3 (*LC3-II*) while significantly suppressing (*p* < 0.05) interleukin-2 (*IL-2*) and mammalian target of rapamycin (*mTOR*). Se had a relieving effect on it, as evidenced by significantly reversing (*p* < 0.05) the changes in the above indicators. Interestingly, Selenoprotein T (*SELENOT*), as a hub membrane-bound selenoprotein, is sensitive to Pb poisoning (Pb exposure decreased *SELENOT* mRNA expression to 56%, 34%, and 19% of the control levels at 30, 60, and 90 days, respectively). Knockdown of *SELENOT* aggravated Pb-induced inflammation, autophagy, and cellular senescence, meaning that *SELENOT* protected against kidney aging via suppressing inflammation and autophagy under Pb stress. These findings establish membrane-bound selenoproteins as essential molecular hubs that orchestrate inflammation resolution and autophagy in Se’s antagonism against Pb-induced kidney aging, highlighting promising therapeutic targets for nephropathy.

## Simple Summary

Lead is an environmental toxicant that damages the kidneys and promotes kidney aging, though the underlying mechanisms are not fully understood. Selenium is an essential nutrient that supports kidney health, mainly through special proteins called selenoproteins. This study explored whether selenium protects chicken kidneys from lead toxicity by focusing on membrane-bound selenoproteins. We treated chickens and kidney cells with lead, with or without selenium, and assessed kidney aging, inflammation, and autophagy. Lead reduced membrane-bound selenoprotein levels and induced kidney aging, while selenium supplementation reversed these effects. Among the proteins studied, Selenoprotein T (*SELENOT*) was the most lead-sensitive and played a key protective role. Reducing *SELENOT* in cells worsened lead-induced damage, confirming its importance in selenium’s protection. These findings highlight membrane-bound selenoproteins, especially *SELENOT*, as potential therapeutic targets for preventing heavy metal-induced kidney injury.

## 1. Introduction

Lead (Pb) is a well-known toxic environmental pollutant. Although measures were taken to control Pb pollution, such as biosorption, phytoremediation, and engineered biochar [1,2], Pb poisoning is still an important health issue all over the world. Pb has been demonstrated to exert toxic effects on the nervous system [3], cardiovascular system [4], liver [5], immune system [6], reproductive and developmental systems [7], as well as the urinary system [8]. Kidney, an organ that maintains a stable internal environment, plays a significant role in clearing metabolic waste and harmful substances from organisms. Excess Pb in the environment poses a threat to kidney health. For instance, in north-central Sri Lanka, Pb levels in rice and vegetables exceeding the permitted intake (3.5 µg/kg BW/day) were associated with an increased incidence of chronic kidney disease [9]. Similarly, Pb-contaminated water in the Agbidiama community of Nigeria was linked to elevated blood Pb levels and impaired kidney function in residents over 20 years of age [10]. The impact of Pb is not limited to humans; wild birds are also affected, as evidenced by Pb accumulation in the feathers, muscles, hearts, livers, and kidneys of sparrow hawks, golden eagles, and white storks in southern Turkey [11]. Moreover, in chickens, a major food source for humans, Pb levels in muscles, brains, hearts, livers, and kidneys from Dhaka, Bangladesh exceeded the Food and Agriculture Organization/World Health Organization (FAO/WHO) maximum allowable limits for meat and viscera [12].

Selenium (Se) is a necessary trace element for organisms. Among human organs, the kidney has the highest Se content. Se is essential for maintaining kidney health in animals. In the absence of Se, tissue lesions occur in pig kidneys [13]. Se exerts its physiological functions mainly through selenoproteins. Among the diverse selenoprotein families, membrane-bound selenoproteins constitute a specialized subgroup that is embedded or anchored in cellular membranes, including the plasma membrane and the membranes of organelles [14,15]. Unlike cytosolic or secreted selenoproteins that primarily function in classic antioxidant defense, membrane-bound selenoproteins are strategically positioned at the interface between intracellular compartments and the extracellular environment, enabling them to participate in critical cellular processes that extend beyond traditional redox regulation. These proteins are involved in maintaining membrane integrity, facilitating protein quality control, and modulating immune responses [14,16,17]. Recent advances have significantly expanded our understanding of the functional repertoire of membrane-bound selenoproteins. For instance, *SELENOT* alleviates inflammation and inhibits senescence mediators in rats with heart failure [18], and also resists cisplatin-induced cardiomyocyte senescence [19]. These findings suggest that membrane-bound selenoproteins may constitute a previously unrecognized molecular link that mediates the protective effects of selenium against Pb toxicity.

It is well-established that aging profoundly affects renal structure and function. The glomerular filtration rate declines progressively with advancing age, and the renal functional reserve becomes limited, thereby increasing the susceptibility of the aged kidney to external insults. Importantly, new research has identified Pb as a potential accelerator of telomere shortening in humans [20]. Pb also accelerates brain aging in rats [21], as well as prefrontal cortex aging in mice [22]. However, whether Pb can accelerate kidney aging remains unknown. Inflammation and autophagy are two fundamental cellular processes critically involved in aging [23,24,25]. Inflammatory response and dysregulation of autophagy have also been implicated in the pathogenesis of multiple renal diseases [26,27]. In the context of Pb exposure, evidence indicates that Pb can induce inflammation [28] and excessive autophagy [29] in kidneys. Therefore, we hypothesize that inflammation and autophagy are involved in the molecular mechanisms of Pb-induced renal aging.

Therefore, the present study was designed to address these critical gaps. What distinguishes this study from previous work is its focus on membrane-bound selenoproteins as an overlooked molecular hub in Se-mediated renal protection, and its identification of Selenoprotein T (*SELENOT*) as the most Pb-sensitive and functionally essential member of this family. Unlike earlier studies that primarily examined the antioxidant functions of cytosolic selenoproteins, this study systematically explored how *SELENOT* integrates inflammatory and autophagic signals to counteract Pb-induced renal aging. To achieve this, we established complementary in vivo and in vitro models, and employed a loss-of-function approach to dissect the role of *SELENOT*. The findings of this study not only advance our understanding of the molecular mechanisms underlying Pb nephrotoxicity, but also position *SELENOT* as a potential therapeutic target for heavy metal-induced kidney diseases.

## 2. Materials and Methods

### 2.1. Animal Model Establishment

The experiment was approved by the Northeast Agricultural University Institutional Animal Care and Use Committee, and the approval number was NEAUEC20250214 (13 March 2025). A total of 180 male Hyline chicks (1-day-old, purchased from Weiwei company in Harbin, China) were provided with a standard diet containing 0.49 mg/kg Se (D) and potable water (W) during the 7-day acclimatization period. The components of D are presented in Table S1. Then, chicks were randomly divided into four groups with 45 chicks each (15 per replicate) using a simple randomization procedure based on a computer-generated random number sequence (the grouping sequence was generated and executed by a researcher who was not involved in the subsequent experiments). The four groups were designated as follows: the control group, the Se group, the Pb group, and the Pb + Se group. The control group was given D and W; the Se group received a diet enriched with Na_2_SeO_3_·5H_2_O-added D (total Se content was 1.00 mg/kg) (SeD) and W; the Pb group offered (CH_3_OO)_2_Pb·3H_2_O-added drinking water (Pb concentration was 350.00 mg/L) (PbW); and the Pb + Se group was supplied with SeD and PbW. In the experiment, the Pb exposure level was established as one-fifth of the LD_50_ for chickens [30], a value that has been adopted in previous studies [31,32,33] and is also consistent with the dosage suggested by Meng (2003) [34]. The Se supplementation dose was selected based on previous studies investigating Se antagonism against Pb toxicity in chickens [32,33]. This dose is well within the safe range for poultry, as dietary Se up to 1.5 mg/kg has been reported to be safe for chicks [35], and no adverse effects were observed with organic Se supplementation at up to 3 mg/kg in laying hens [36]. (CH_3_OO)_2_Pb·3H_2_O and Na_2_SeO_3_·5H_2_O were analytical reagent grade and were purchased from Tianjinzhiyuan Chemical Reagent Co., Ltd., Tianjin, China. This number of chickens was also considered sufficient to detect biologically meaningful differences in key parameters based on the laboratory’s prior experience with similar chicken models. The chickens were provided food and water ad libitum, and were raised under the same conditions as recorded in Li et al. (2024) [37] at the Laboratory Animal Center, Animal Medical College, Northeast Agricultural University (Harbin, China) until the end of the experiment. Animals were monitored twice daily (morning and evening) throughout the 90-day experimental period, including weekends and holidays, by trained personnel who were familiar with the clinical signs of Pb toxicity in poultry.

### 2.2. Sample Collection

On days 30, 60, and 90 of the experiment, respectively, 15 chickens from each group were euthanized. A single dose of pentobarbital sodium (300 mg/kg) was rapidly administered via the wing vein to achieve prompt euthanasia [38]. Then, the kidneys were immediately separated and cleaned with ice-cold saline. Five chickens were randomly selected as independent biological replicates (*n* = 5) for quantitative measurements, including qRT-PCR and oxidative stress indicators. The remaining ten chickens were used for histopathological examination and tissue storage. The random selection of the five chickens was performed by a researcher who was not involved in the subsequent experiments, using a computer-generated random number sequence, thereby minimizing selection bias.

### 2.3. Microstructural Observation

Briefly, the kidney tissues fixed with paraformaldehyde solution were dehydrated in gradient alcohol (30, 50, 70, 90, and 100%), embedded in paraffin, and sectioned to nominal thicknesses of 4 μm. The sections were stained with hematoxylin and eosin. Finally, the sections were subjected to microscopic examination (Eclipse 80i, Nikon, Tokyo, Japan) and photographs were taken.

### 2.4. Ultrastructural Observation

The tissue blocks were taken out from 2.5% glutaraldehyde phosphate buffer saline, rinsed with 0.1 mol/L PBS, put in 1% osmium tetroxide at 4 °C for 1 h, and rinsed again with 0.1 mol/L PBS. Ethanol was used to soak tissue blocks, and then epoxy resin was used to embed tissue blocks. After that, an ultramicrotome (LR604, RMC company, Yorba Linda, CA, USA) was used to slice the tissue blocks. The obtained sections were stained with uranyl acetate and lead citrate. The ultrastructures of the chicken kidneys were observed and photographed using a transmission electron microscope (Model JEM-1200EX, Jeol Jem, Tokyo, Japan).

### 2.5. Oxidative Stress Indicator Detection

Kidney tissue homogenate solution was centrifuged at 16,000 *g* and 4 °C for 5 min. The obtained supernatant was used for determining total antioxidant capacity (T-AOC), glutathione s-transferase (GST), and catalase (CAT) activities and hydrogen peroxide (H_2_O_2_) content in chicken kidneys using kits produced by Nanjing Jiancheng Bioengineering Institute (Nanjing, China). Kit numbers were listed as follows: A015-1-2 (T-AOC assay kit), A004-1-1 (GST assay kit), A007-1-1 (CAT assay kit), and A064-1-1 (H_2_O_2_ assay kit). Operation steps were in accordance with the instructions provided by the manufacturer. Briefly, for the T-AOC assay, the supernatant was mixed with the provided substrate solution and incubated at 37 °C for 30 min, followed by absorbance measurement at 520 nm. For GST activity, the supernatant was incubated with the provided substrate solution at 37 °C for 30 min, and 20 μmol/L reduced glutathione was added. The absorbance at 412 nm was recorded. For CAT activity, the supernatant was reacted with H_2_O_2_ at 37 °C for 1 min. The reaction was rapidly terminated by the addition of ammonium molybdate, and the remaining H_2_O_2_ reacted with ammonium molybdate to form a yellowish complex, which was then measured at 405 nm. For H_2_O_2_ content, the supernatant was mixed with the chromogenic reagent provided in the kit, followed by absorbance measurement at 405 nm.

### 2.6. Human Kidney-2 (HK-2) Cell Culture

HK-2 cells purchased from Shanghai Fuheng Biotechnology Co., Ltd. (Shanghai, China) were maintained in DMEM/F-12 (Gibco, Brooklyn, NY, USA) supplemented with 10% fetal bovine serum (FBS, Biological Industries, Israel), 100 U/mL penicillin, and 100 μg/mL streptomycin (Biological Industries, Beit HaEmek, Israel). Culture flasks were placed in a humidified incubator set at 37 °C with a 5% CO_2_ atmosphere. Routine subculturing was performed when cell monolayers reached approximately 80–90% confluence. The spent medium was discarded, and the adherent cells were gently rinsed with pre-warmed phosphate-buffered saline (PBS, pH 7.4) to remove residual serum. A 0.25% trypsin-EDTA solution (Gibco, USA) was added to the flask (approximately 1 mL for a T25 flask). Detachment was verified under an inverted microscope. Once rounding and separation occurred, complete growth medium containing serum was added to neutralize trypsin activity. The cell suspension was gently pipetted to break up clumps, and the cells were reseeded into new flasks at a split ratio of 1:2. Medium was renewed every 2 days.

### 2.7. Cell Counting Kit-8 (CCk-8) Assay for Cell Viability

HK-2 cells were seeded into 96-well plates and incubated overnight to allow attachment. The culture medium was then replaced with fresh medium containing different concentrations of Pb(CH_3_OO)_2_ or Na_2_SeO_3_, and the cells were further incubated for 24 h. Based on the studies by Tian et al. [39] and Qi et al. [40], the concentrations of Pb(CH_3_COO)_2_ for treating HK-2 cells were set to 0, 25, 50, 75, 100, 125, 150, 175, 200, 225, 250, 275, and 300 μM, and the concentrations of Se for treating HK-2 cells were set to 0, 0.5, 1, 1.5, 2, 2.5, 3, 3.5, 4, 4.5, 5, 5.5, 6, and 6.5 μM. Then, 10 μL of Cell Counting Kit-8 solution (Dojindo, Kumamoto, Japan) was added to each well, followed by incubation at 37 °C for 1.5 h. Absorbance at 450 nm was recorded using a microplate reader (Thermo Fisher Scientific, Waltham, MA, USA).

### 2.8. The Knockdown of SELENOT

Transfection with specific siRNAs was used to suppress *SELENOT* expression. The siRNAs were custom-designed and synthesized by Chengxin Biotechnology Co., Ltd. (Shanghai, China). Prior to transfection, HK-2 cells were seeded in 6-well plates and grown until they reached approximately 70% confluence. The culture medium was then replaced, and transfection was performed for 24 h using Opti-MEM medium and Lipofectamine (both Thermo Fisher Scientific, USA) in the presence of either negative control siRNA (NC-si) or SELENOT-targeting siRNA, according to the manufacturer’s instructions.

### 2.9. Immunofluorescence Detection of Autophagy-Related Protein

Cells were cultured on sterile coverslips placed in 6-well plates. After the assigned 24-h treatment, the culture medium was removed, and the cells were rinsed three times with PBS. Fixation was performed with 4% paraformaldehyde for 10 min, followed by three additional PBS washes. The cells were then blocked with 5% BSA for 30 min. The blocking solution was discarded, and the coverslips were incubated overnight at 4 °C with an anti-p62 primary antibody (1:200 dilution). After recovery of the primary antibody, the cells were probed with a fluorophore-conjugated secondary antibody for 1 h at room temperature and washed three times with PBS. Nuclei were counterstained with DAPI staining solution for 1 min and rinsed three times with PBS. Fluorescence images were taken using a Nikon fluorescence microscope (Nikon Corporation, Tokyo, Japan), and the fluorescence signal intensity was measured using ImageJ software (version 1.53).

### 2.10. Relative mRNA Expression Analysis

#### 2.10.1. Primer Synthesis

Primer sequences and GenBank (www.ncbi.nlm.nih.gov/genbank/ accessed on 5 March 2026) accession numbers of the detected genes are listed in Table S2 (for chicken kidneys) and Table S3 (for HK-2 cells). GAPDH served as the internal reference gene. All primers were synthesized by Invitrogen Biotechnology Co., Ltd. (Shanghai, China).

#### 2.10.2. Total RNA Isolation and Reverse Transcription

Total RNA was extracted from kidney tissues frozen at −80 °C with TRIzol reagent following the method provided by the manufacturer (Invitrogen, China). A spectrophotometer (Healthcare Bio-Sciences AB, Uppsala, Sweden) was used to determine the RNA purity. The OD_260_/OD_280_ ratio was between 1.8 and 2.1 for all samples, meeting the experimental requirements. Complementary DNA (cDNA) was synthesized using the PrimeScript™ RT Reagent Kit (TaKaRa, Shiga, Japan) in a volume of 60 µL (containing 5 µg of the total RNA) according to the manufacturer’s instructions. Obtained cDNA was diluted with DEPC-treated water and stored at −20 °C until use.

#### 2.10.3. Quantitative Real-Time PCR

Quantitative real-time PCR was performed using a LightCycler^®^ 96 real-time fluorescence quantitative PCR instrument (Roche, Basel, Switzerland) with the SYBR^®^ PrimeScript^TM^ RT-PCR Kit (Roche, Switzerland) following the manufacturer’s instructions. The reaction system comprised 0.3 μL of forward primer (10 μM), 0.3 μL of reverse primer (10 μM), 1 μL of diluted cDNA, 3.4 μL of sterile distilled water, and 5 μL of 2 × SYBR green PCR master mix (Takara, Beijing, China). The PCR procedure was as follows: at 52 °C for 2 min and at 95 °C for 10 min; followed by 40 cycles of amplification and quantification at 95 °C for 15 s, at 60 °C for 60 s, and at 95 °C for 15 s; and at 60 °C for 20 s. Each sample was run in triplicate. Relative mRNA expression was calculated according to the 2^−ΔΔCT^ method.

### 2.11. Statistical Analysis

All experimental data were presented as mean ± standard deviation (SD). One-way and two-way analyses of variance (ANOVA) were performed using SPSS (version 25.0, SPSS Inc., Chicago, IL, USA). Normality was evaluated using the Shapiro–Wilk test (α = 0.05). Homogeneity of variances was assessed using Levene’s test (α = 0.05). In all cases, the assumptions of normality (*p* > 0.05 for Shapiro–Wilk) and homogeneity of variance (*p* > 0.05 for Levene) were satisfied for the data analyzed in this study. For all parametric tests, a two-way analysis of variance (ANOVA) was used to evaluate the effects of treatment (control, Se, Pb, Pb + Se), time (30, 60, 90 days), and their interaction, followed by Tukey’s honest significant difference (HSD) post hoc test for multiple comparisons among groups at the same time point, as well as between different time points within the same group. For the in vitro experiments (HK-2 cells), which involved only a single time point, one-way ANOVA followed by Tukey’s HSD test was applied. Prism 6.0 (Graph-Pad Software, USA) was used to draw bar charts. Statistical significance was assigned as *p* < 0.05.

## 3. Results

### 3.1. Se Relived Pb-Caused Chicken Kidney Damage

**Figure 1:**
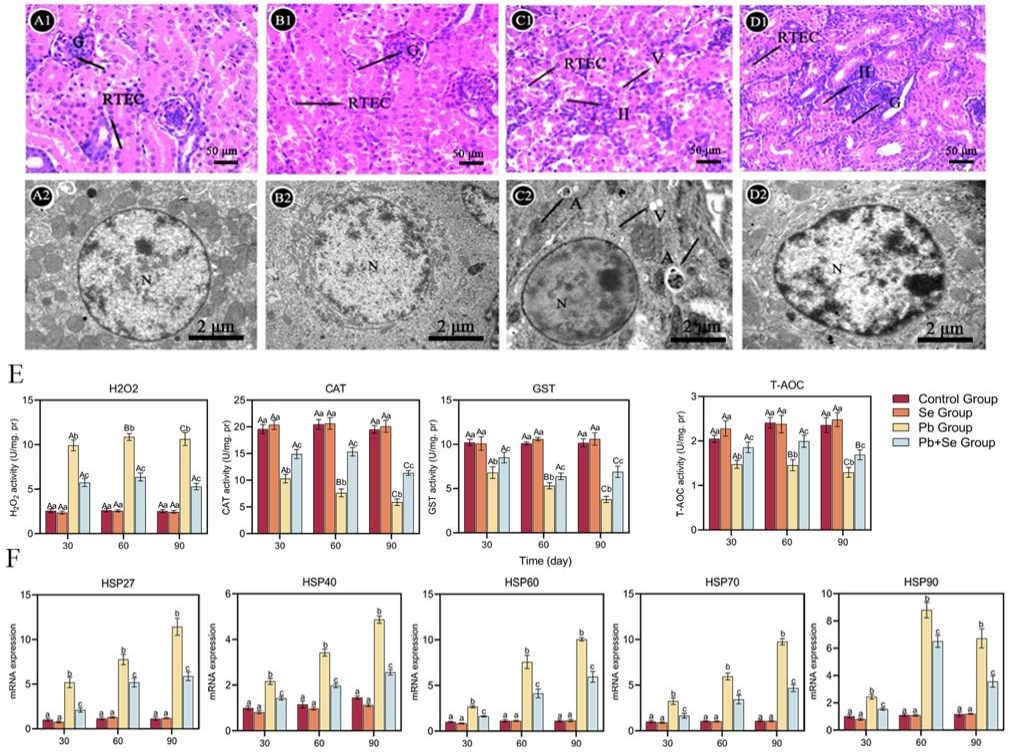
Effects of Pb, Se, and their co-treatment on chicken kidneys. (**A1**–**D1**) Paraffin sections of kidney tissues stained with hematoxylin–eosin (400×). The control (**A1**) and Se-alone (**B1**) groups show clear glomerular structure and distinct capsular spaces. The Pb-alone group (**C1**) exhibits glomerular swelling, blurred boundaries of renal cysts, swollen tubular epithelial cells, extensive inflammatory infiltration, and vacuolization. These pathological injuries are markedly alleviated in the Pb + Se group (**D1**). (**A2**–**D2**) Ultrathin sections stained with uranyl acetate and lead citrate (12,000×). Normal organelle morphology is seen in the control (**A2**) and Se-alone (**B2**) groups. The Pb-alone group (**C2**) shows hyperchromatic nuclei and visible autophagosomes, whereas the Pb + Se group (**D2**) shows reduced ultrastructural damage. G: glomerulus; RTEC: renal tubular epithelial cell; II: inflammatory infiltration; V: vacuole; N: nucleus; A: autophagosome. (**E**): Oxidative stress indicators, including H_2_O_2_ content, CAT, GST, and T-AOC activities. (**F**): mRNA expressions of *HSPs*, including *HSP27*, *HSP40*, *HSP60*, *HSP70*, and *HSP90*. At the same time point, bars with different lowercase letters represent statistically significant (*p* < 0.05) differences between the groups. In the same group, bars with different uppercase letters represent statistically significant (*p* < 0.05) differences at the different time points.

To explore the effect of Pb on chicken kidneys and the mitigative effect of Se on Pb poisoning, microstructural and ultrastructural observation, as well as the detection of oxidative stress indicators and HSPs, were performed. Histological alterations of chicken kidneys are shown in Figure 1 on day 90. In the control (Figure 1(A1)) and Se (Figure 1(B1)) groups, the glomerular structure was clear and glomerular cavity was clearly visible. In the Pb group (Figure 1(C1)), the glomerulus was swollen, the boundaries of renal cysts were unclear, renal tubular epithelial cells were swollen, inflammatory cells infiltrated extensively, and vacuolarization occurred compared with the control group. In the Pb + Se group (Figure 1(D1)), the glomeruli were slightly swollen, the margins of the renal cysts were a little blurred, renal tubular epithelial cells were slightly swollen, and inflammatory infiltration decreased compared with the Pb group. Ultrastructural results of chicken kidneys were as follows. Organelles were normal in the control (Figure 1(A2)) and Se (Figure 1(B2)) groups. In the Pb group (Figure 1(C2)), the nuclei was hyperchromatic, and autophagosomes were visible after Pb treatment for 90 days; these changes were relieved in the Pb + Se group (Figure 1(D2)).

Oxidative stress indicators (Figure 1E) and heat shock proteins (HSPs) (Figure 1F) are indicative of the stress level in tissues. We found that Pb decreased T-AOC, GST, and CAT, increased H_2_O_2_, heat shock protein *(HSP)27*, *HSP40*, *HSP60*, *HSP70*, and *HSP90* at all three time points, supporting that Pb caused kidney injury. Se supplementation reversed the above changes in these factors. All findings indicate that Se can alleviate Pb-induced kidney damage, and that inflammation, autophagy, and cellular senescence may be the potential mechanisms.

### 3.2. Se Ameliorated Kidney Aging Caused by Pb in Chickens

**Figure 2:**
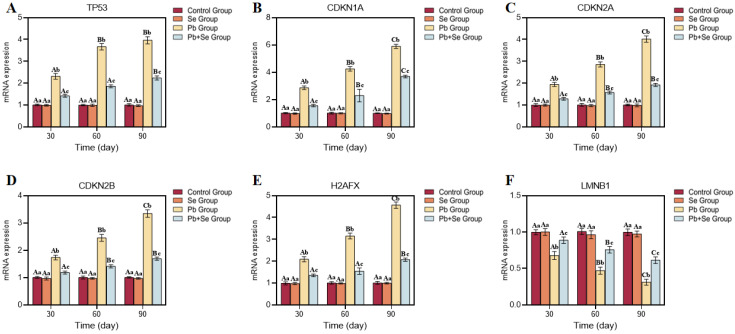
Effects of Pb, Se, and their co-treatment on the mRNA expression of senescence-related marker genes in chicken kidneys. Relative mRNA levels of *TP53* (**A**), *CDKN1A* (**B**), *CDKN2A* (**C**), *CDKN2B* (**D**), *H2AFX* (**E**), and *LMNB1* (**F**) were measured in the control, Se, Pb, and Pb + Se groups at 30, 60, and 90 days. Bars represent mean ± SD (*n* = 5/group). At the same time point, bars with different lowercase letters represent statistically significant (*p* < 0.05) differences between the groups. In the same group, bars with different uppercase letters represent statistically significant (*p* < 0.05) differences at the different time points.

Heavy metal-induced cellular senescence has been reported in previous studies. Six cellular senescence marker genes, including tumor protein P53 (*TP53*) (Figure 2A), cyclin dependent kinase inhibitor *(CDKN)1A* (Figure 2B), *CDKN2A* (Figure 2C), *CDKN2B* (Figure 2D), H2A histone family member X (*H2AFX*) (Figure 2E), and lamin B1 (*LMNB1*) (Figure 2F) were examined to investigate whether Pb induces kidney senescence in chickens and whether Se exerts an anti-senescence effect. Compared with the control group, the Se group showed no significant changes in *TP53*, *CDKN1A*, *CDKN2A*, *CDKN2B*, *H2AFX*, or *LMNB1*. At all three Pb exposure time points, *TP53*, *CDKN1A*, *CDKN2A*, *CDKN2B*, and *H2AFX* were increased, while *LMNB1* was decreased, indicating that Pb accelerates kidney senescence in chickens. Se supplementation reversed the Pb-induced changes, demonstrating that Se effectively protects against Pb-induced kidney senescence.

### 3.3. Inflammation and Autophagy Partook in Kidney Aging in the Case of Se/Pb Treatment

**Figure 3:**
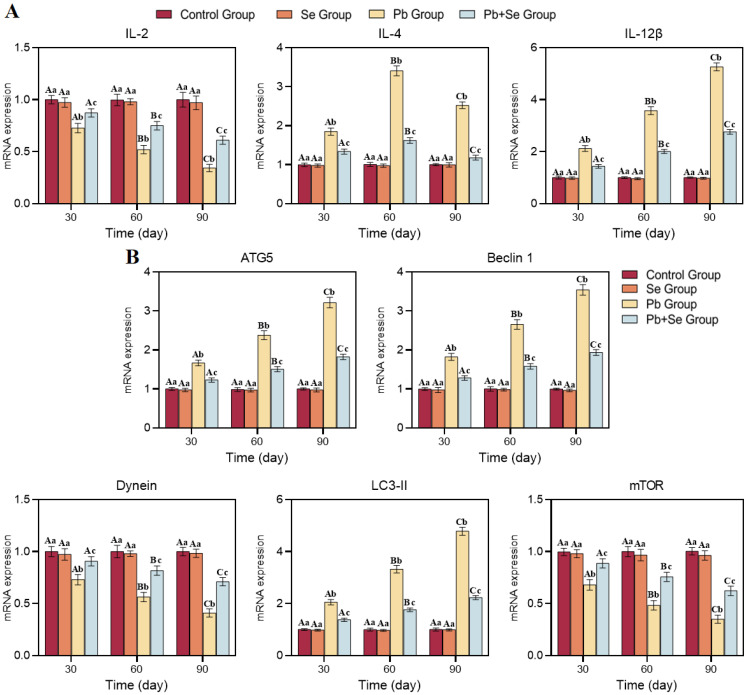
Effects of Pb, Se, and their co-treatment on the expression of inflammation- and autophagy-related genes in chicken kidneys. (**A**) mRNA levels of inflammatory genes *IL-2*, *IL-4*, and *IL-12β*. (**B**) mRNA levels of autophagy-related genes *ATG5*, *Beclin 1*, *Dynein*, *LC3-II*, and *mTOR*. Bars represent mean ± SD (*n* = 5/group). At the same time point, bars with different lowercase letters represent statistically significant (*p* < 0.05) differences between the groups. In the same group, bars with different uppercase letters represent statistically significant (*p* < 0.05) differences at the different time points.

Inflammatory response and dysregulated autophagy are two mechanisms of cellular senescence. Inflammatory-related genes (*interleukin (IL)-2*, *IL-4*, and *IL-12β*) and autophagy genes (*autophagy-related genes autophagy related 5 (ATG5)*, *BCL2 interacting coiled coil protein 1 (Beclin 1)*, *Dynein*, *microtubule-associated protein 1A/1B-light chain 3 (LC3-II)*, and *mammalian target of rapamycin (mTOR)*) were examined to investigate the mechanism of Se relieving Pb-induced kidney aging. Regarding to inflammatory-related genes (Figure 3A), no significant difference was found between the Se group and the control group. There was a significant decrease in *IL-2*, and conversely, a significant increase in *IL-4* and *IL-12β* in the Pb group compared with those in the Se and control groups. An upregulation of *IL-2* in the Pb + Se group was found compared with the Pb group, on the contrary, a downregulation of *IL-4* and *IL-12β* in the Pb + Se group was found compared with the Pb group. In the Pb group, IL-2 mRNA expression was reduced, and *IL-4* and *IL-12β* mRNA expression was elevated with the increase in Pb treatment duration in chicken kidneys. Regarding the autophagy genes (Figure 3B), the changes of *ATG5*, *Beclin 1*, *Dynein*, *LC3-I*, and *LC3-II* showed a similar trend to *IL-4* and *IL-12β*, while the change of mTOR was the same as IL-2. All of the above data indicate that Pb caused kidney aging with the involvement of inflammatory response and excessive autophagy.

### 3.4. Membrane-Bound Selenoproteins Participate in the Mechanism of Se Alleviating Pb Toxicity, in Which SELENOT Is the Most Sensitive and Serves as a Hub Protein

**Figure 4:**
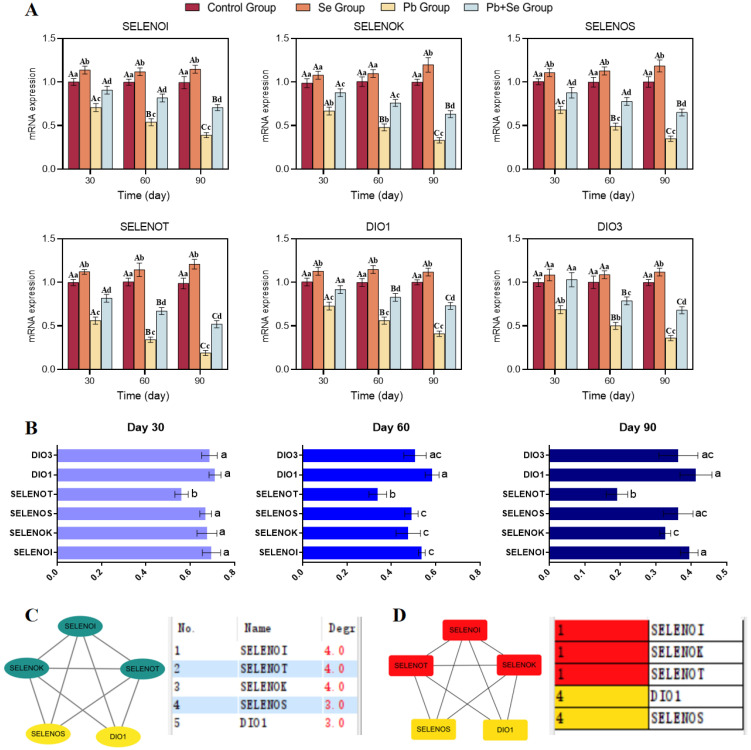
Involvement of membrane-bound selenoproteins in Se-mediated protection against Pb toxicity and the identification of SELENOT as a hub protein. (**A**) mRNA expression levels of six membrane-bound selenoproteins (*SELENOI*, *SELENOK*, *SELENOS*, *SELENOT*, *DIO1*, and *DIO3*) in the control, Se, Pb, and Pb + Se groups at 30, 60, and 90 days. (**B**) Comparison of the magnitude of changes in each selenoprotein under Pb exposure, showing that *SELENOT* exhibits the greatest sensitivity. (**C**) PPI network core protein ranking based on degree centrality. Green indicates the top 3 selenoproteins, and yellow indicates the 4th and 5th ranked selenoproteins. (**D**) PPI network core protein ranking based on the MCC algorithm. Red indicates the top 3 selenoproteins, and yellow indicates the 4th and 5th ranked selenoproteins. Bars represent mean ± SD (*n* = 5/group). At the same time point, bars with different lowercase letters represent statistically significant (*p* < 0.05) differences between the groups. In the same group, bars with different uppercase letters represent statistically significant (*p* < 0.05) differences at the different time points.

Since Se exerts its biological functions mainly through selenoproteins, six membrane-bound selenoproteins: *selenoprotein (SELENO)I*, *SELENOK*, *SELENOS*, *SELENOT*, *iodothyronine deiodinase (DIO)1*, and *DIO3* (Figure 4A) were detected in the study. The results showed that all six selenoproteins were higher in the Se group than in the control group, indicating that the Se supplemented in the diet was utilized by the organism. Under Pb exposure, *SELENOI*, *SELENOK*, *SELENOS*, *SELENOT*, *DIO1*, and *DIO3* were decreased at all three experimental time points, and Se supplementation reversed this phenomenon, suggesting that membrane-bound selenoproteins are involved in the mechanism by which Se mitigates Pb-induced renal toxicity. Notably, when comparing the changes in each membrane-bound selenoprotein under Pb exposure, *SELENOT* showed the greatest change (Figure 4B), indicating that *SELENOT* is the most Pb-sensitive membrane-bound selenoprotein in the kidney. Protein-protein interaction (PPI) network analysis can identify core proteins, i.e., key nodes in the interaction network of membrane-bound selenoproteins. Two ranking methods, degree value and MCC, were included. Degree value refers to the number of direct connections a protein has with other proteins in the network, enabling the preliminary screening and ranking of proteins. The MCC method better identifies the core status of a protein within a fine-grained subnetwork. Using both degree value (Figure 4C) and maximal clique centrality (MCC) (Figure 4D) methods for PPI network analysis, SELENOT consistently ranked first together with SELENOI and SELENOK was found, indicating that SELENOT is not only sensitive to Pb toxicity, but also a membrane-bound selenoprotein with potential core functions.

### 3.5. Se Alleviated Pb-Induced Selenoprotein Inhibition and Cellular Senescence Involving Inflammation and Autophagy In Vitro

**Figure 5:**
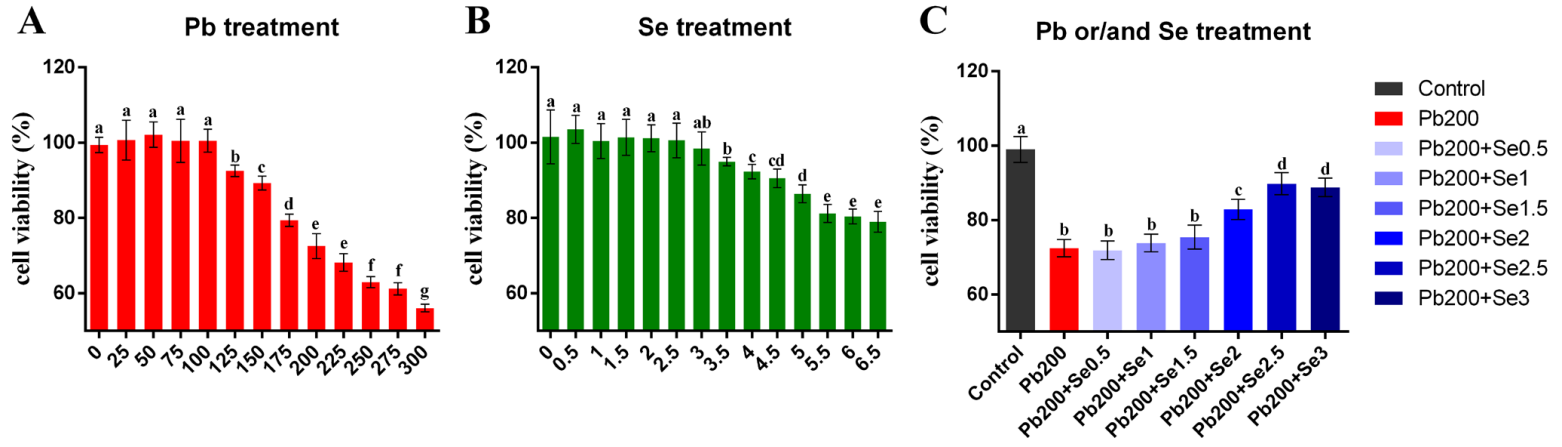
Effects of different concentrations of Pb and Se on HK-2 cell viability and the selection of Se intervention concentration. (**A**) Cell viability after 24 h treatment with Pb(CH_3_COO)_2_ at concentrations of 0–300 μM. A concentration of 200 μM (viability 72.53%) was chosen for subsequent Pb exposure. (**B**) Cell viability after 24 h treatment with Na_2_SeO_3_ at 0–6.5 μM. (**C**) Recovery of cell viability under 200 μM Pb exposure with co-treatment of Se at 0.5–3 μM. Se at 2.5 μM provided the highest protective effect and was selected for further experiments. Bars represent mean ± SD (*n* = 4/group). Bars with different lowercase letters represent statistically significant (*p* < 0.05) differences between the groups.

An HK-2 cell Pb and/or Se treatment model was established to verify the molecular mechanism of Se in alleviating Pb toxicity in vitro. First, HK-2 cells were treated with different concentrations of Pb, and cell viability was measured to screen for the appropriate Pb concentration for Pb exposure experiments (Figure 5A). At Pb concentrations of 25–100 μM, cell viability showed no significant difference compared with the control group; at 125 μM and higher concentrations, cell viability was lower than that of the control group and decreased with increasing Pb concentration. Therefore, 200 μM (cell viability 72.53%) was selected as the Pb treatment concentration. Subsequently, HK-2 cells were treated with various concentrations of Se to identify Se concentrations that could alleviate Pb toxicity (Figure 5B). At Se concentrations of 0.5–3 μM, cell viability was not significantly different from the control group. At 3.5 μM and higher, cell viability was lower than that of the control group and decreased with increasing Se concentration. Six Se concentrations (0.5, 1, 1.5, 2, 2.5, and 3 μM) were chosen to alleviate Pb toxicity. Se at 0.5–1.5 μM was found to be ineffective in reversing Pb-induced cytotoxicity. Se at 2, 2.5, and 3 μM significantly increased cell viability under Pb exposure, with the highest viability observed at 2.5 μM Se treatment (Figure 5C); therefore, 2.5 μM was selected as the experimental Se concentration.

**Figure 6:**
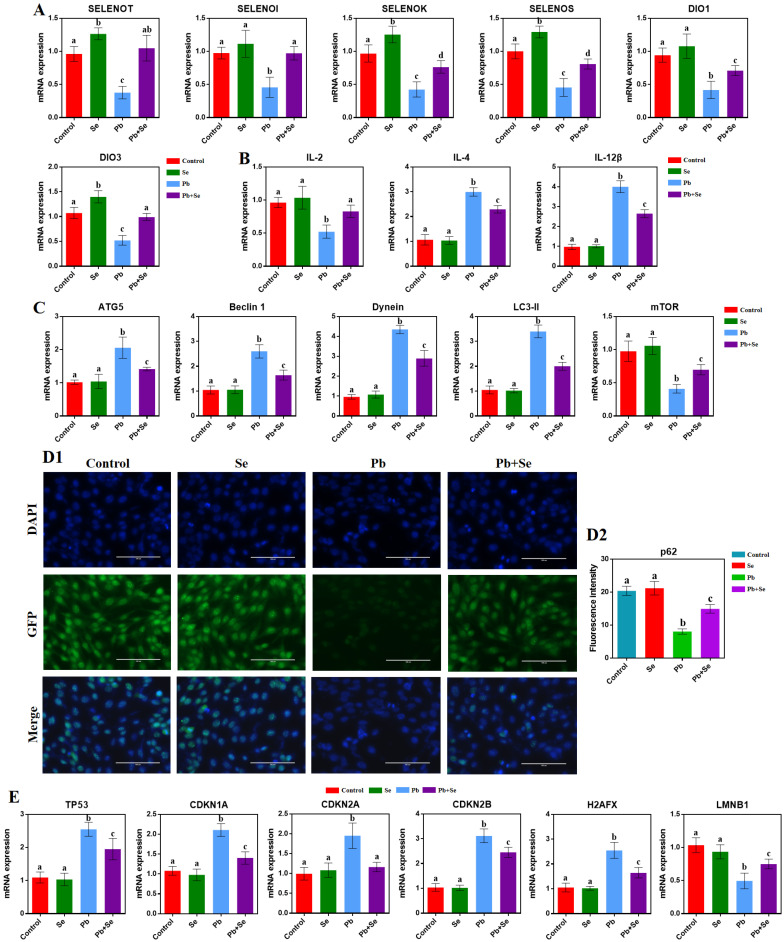
Verification of the molecular mechanism in HK-2 cells: effects of Pb, Se, and their co-treatment on selenoprotein expression, inflammation, autophagy, and senescence markers (in vitro). (**A**) mRNA levels of six membrane-bound selenoproteins (*SELENOI*, *SELENOK*, *SELENOS*, *SELENOT*, *DIO1*, *DIO3*). (**B**) mRNA levels of inflammatory genes (*IL-2*, *IL-4*, *IL-12β*). (**C**) mRNA levels of autophagy genes (*ATG5*, *Beclin 1*, *Dynein*, *LC3-II*, *mTOR*). (**D1**,**D2**) Immunofluorescence detection of the p62 protein (with green fluorescence) with DAPI nuclear counterstain (with blue fluorescence) and corresponding fluorescence intensity quantification. (**E**) mRNA levels of senescence markers (*TP53*, *CDKN1A*, *CDKN2A*, *CDKN2B*, *H2AFX*, *LMNB1*). Bars represent mean ± SD (*n* = 4/group). Bars with different lowercase letters represent statistically significant (*p* < 0.05) differences between the groups.

Based on the selected Pb exposure concentration and Se intervention concentration, the expression of six membrane-bound selenoproteins (including *SELENOI*, *SELENOK*, *SELENOS*, *SELENOT*, *DIO1*, and *DIO3*, shown in Figure 6A), three inflammation-related genes (including *IL-2*, *IL-4*, and *IL-12β*, shown in Figure 6B), five autophagy-related genes (including *ATG5*, *Beclin 1*, *Dynein*, *LC3-II*, and *mTOR*, shown in Figure 6C), the autophagy-related protein p62 (Figure 6(D1,D2)), and six senescence marker genes (including *TP53*, *CDKN1A*, *CDKN2A*, *CDKN2B*, *H2AFX*, and *LMNB1*, shown in Figure 6E) were measured under Pb and/or Se treatment. Consistent with the in vivo findings, the six membrane-bound selenoproteins were increased in the Se group compared with the control group, whereas the other factors showed no significant changes relative to the control group. Under Pb exposure, *SELENOI*, *SELENOK*, *SELENOS*, *SELENOT*, *DIO1*, *DIO3*, *IL-2*, *mTOR*, p62, and *LMNB1* were decreased, while *IL-4*, *IL-12β*, *ATG5*, *Beclin 1*, *Dynein*, *LC3-II*, *LC3*, *TP53*, *CDKN1A*, *CDKN2A*, *CDKN2B*, and *H2AFX* were increased. Se supplementation reversed the above changes. These results further confirm that Se alleviates Pb-induced decreases in membrane-bound selenoproteins, inflammation, autophagy, and kidney senescence.

### 3.6. SELENOT Exerts an Anti-Renal Aging Effect by Regulating Inflammation and Autophagy

**Figure 7:**
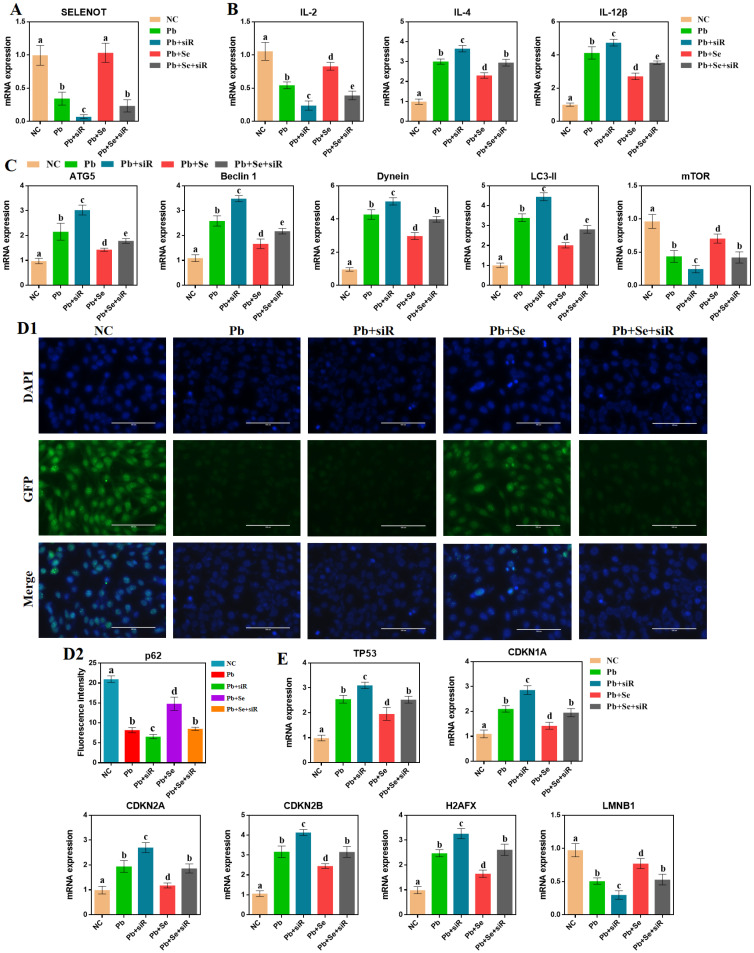
Effects of SELENOT knockdown on the expression of selenoprotein, inflammation, autophagy, and senescence markers in HK-2 cells under Pb and/or Se treatment. (**A**) mRNA level of *SELENOT* after transfection with NC-siRNA or si-SELENOT in the Pb and Pb + Se groups, confirming successful knockdown. (**B**) mRNA levels of inflammatory genes (*IL-2*, *IL-4*, *IL-12β*). (**C**) mRNA levels of autophagy genes (*ATG5*, *Beclin 1*, *Dynein*, *LC3-II*, *mTOR*). (**D1**,**D2**) Immunofluorescence detection of the p62 protein (with green fluorescence) with DAPI nuclear counterstain (with blue fluorescence) and corresponding fluorescence intensity quantification. (**E**) mRNA levels of senescence markers (*TP53*, *CDKN1A*, *CDKN2A*, *CDKN2B*, *H2AFX*, *LMNB1*). Bars represent mean ± SD (*n* = 4/group). Bars with different lowercase letters represent statistically significant (*p* < 0.05) differences between the groups.

To further explore the role of *SELENOT* in Se-mediated alleviation of Pb-induced cellular senescence, *SELENOT* was knocked down in HK-2 cells. After establishing the *SELENOT* knockdown model, SELENOT levels were substantially reduced in HK-2 cells under both Pb and Pb + Se treatments (Figure 7A), indicating successful *SELENOT* knockdown. The detection of inflammatory genes (Figure 7B) showed that *SELENOT* knockdown increased *IL-2*, *IL-4*, and *IL-12β* under both Pb and Pb + Se treatments, indicating that *SELENOT* knockdown weakened the anti-inflammatory effect of Se. The detection of autophagy-related genes (Figure 7C) and autophagy protein (Figure 7(D1,D2)) showed that *SELENOT* knockdown increased the mRNA levels of *ATG5*, *Beclin 1*, *Dynein*, *LC3-I*, and *LC3-II* while decreasing the mRNA level of *mTOR* and the protein level of p62 under both Pb and Pb + Se treatments, indicating that *SELENOT* knockdown weakened the effect of Se in alleviating excessive autophagy. As expected, under Pb and Pb + Se treatments, the cellular senescence genes *TP53*, *CDKN1A*, *CDKN2A*, *CDKN2B*, and *H2AFX* were increased by *SELENOT* knockdown, whereas *LMNB1* was decreased by *SELENOT* knockdown (Figure 7E). Together, these data demonstrate that *SELENOT* alleviates cellular senescence by modulating inflammation and excessive autophagy.

## 4. Discussion

Chronic Pb exposure remains a persistent environmental threat, with the kidney being one of its primary target organs due to its role in the filtration and accumulation of xenobiotics. Despite growing evidence linking Pb to multisystemic toxicity, the specific mechanisms by which Pb accelerates renal aging have remained elusive. Se, an essential trace element, exerts its biological functions predominantly through selenoproteins. Among these, membrane-bound selenoproteins, strategically localized at the endoplasmic reticulum and plasma membrane, are increasingly recognized as key regulators of cellular homeostasis, yet their role in mediating Se’s protection against Pb-induced renal aging has not been systematically explored. In this study, the findings provide the first evidence that Pb exposure downregulates membrane-bound selenoproteins, induces renal aging, and triggers inflammatory and autophagic responses in chicken kidneys. Importantly, *SELENOT* is identified as a hub membrane-bound selenoprotein that is highly sensitive to Pb and functionally required for Se’s protective effects. The findings position *SELENOT* as a critical molecular switch linking Se status to inflammation-autophagy crosstalk in the context of Pb-induced renal aging.

### 4.1. Pb Induces Renal Aging, Inflammation, and Autophagy: A Pathological Triad

Histological and ultrastructural analyses revealed that Pb exposure caused severe renal damage, including glomerular swelling, tubular epithelial vacuolization, inflammatory infiltration, and autophagosomal accumulation. These pathological features were accompanied by a marked decline in antioxidant capacity (decreased T-AOC, GST, CAT) and elevated H_2_O_2_ levels, confirming oxidative stress as an early event. *IL-2* is an anti-inflammatory factor. Subcutaneous injection of *IL-2* alleviated inflammatory damage in the kidneys of mice with systemic lupus erythematosus [41]. *IL-4* and IL-12β are pro-inflammatory mediators. Mice with over-expressed IL-4 in lungs developed lung inflammatory injury [42]. After *IL-12β* was knocked out in mice with spinal cord impairment, spinal cord inflammatory damage was relieved [43]. Concurrently, Pb upregulated pro-inflammatory cytokines (*IL-4*, *IL-12β*) and suppressed *IL-2*, indicative of a shift toward a pro-inflammatory milieu. *Beclin 1* is an important autophagy promoting gene. Under stress, *mTOR* can be inhibited and autophagy can be promoted in cells [44]. *ATG5*, a core autophagy protein, functions in regulating the formation of autophagosomes and is considered to be indispensable for autophagy [45]. *LC3-I* participates in the production of *LC3-II*. *LC3-II* is an autophagy marker and can be recruited to autophagosome membranes. *Dynein* is involved in the fusion of autophagosomes and lysosomes [46]. *Beclin 1*, *ATG5*, *LC3-II*, and *Dynein* were elevated, while *mTOR* was suppressed, suggesting excessive autophagic flux. Previous studies supported these findings. Bisphenol A induced autophagy with increased *Beclin 1* and *LC3-II* in mouse ovaries [47]. Pb increased mRNA and protein levels of *Beclin 1*, *ATG 5*, *LC3-I*, *LC3-II*, and *Dynein*; decreased mRNA and protein levels of *mTOR*; and induced autophagy in chicken spleens [48]. Fluoride caused inflammatory injury with elevated *IL-4* in rat livers and kidneys [49]. Decreased *IL-2* and increased *IL-4* and *IL-12β* were observed in Pb-induced chicken testicular inflammatory injury [31]. These results collectively demonstrate that Pb triggers a triad of oxidative stress, inflammation, and autophagy, which together drive renal aging. Importantly, the time-dependent progression of these changes underscores the chronic, cumulative nature of Pb nephrotoxicity.

### 4.2. Membrane-Bound Selenoproteins: A Previously Overlooked Molecular Hub

Se’s protective effects are mediated through selenoproteins, but the specific contribution of membrane-bound selenoproteins in renal protection has been underexplored. Our study fills this gap by systematically profiling six membrane-bound selenoproteins (*SELENOI*, *SELENOK*, *SELENOS*, *SELENOT*, *DIO1*, *DIO3*) under Pb and Se interventions. Pb uniformly suppressed their expression across the three time points, while Se supplementation restored their expression, indicating that these proteins are not only responsive to Se status, but also vulnerable to Pb toxicity. Strikingly, *SELENOT* exhibited the greatest magnitude of change under Pb exposure, suggesting exceptional sensitivity. Furthermore, PPI network analysis, using both degree centrality and MCC algorithms, ranked *SELENOT* alongside *SELENOI* and *SELENOK* as the top hub protein. This positions *SELENOT* as a central node in the membrane-bound selenoprotein interaction network, capable of influencing broader cellular pathways.

### 4.3. SELENOT as a Key Effector Against Pb-Induced Renal Aging via Inflammation and Autophagy

To dissect the functional role of *SELENOT*, a loss-of-function approach in HK-2 cells was employed. Knockdown of *SELENOT* exacerbated Pb-induced inflammation (further increased *IL-2*, *IL-4*, *IL-12β*), aggravated autophagic dysregulation (elevated *ATG5*, *Beclin 1*, *LC3-II*, *Dynein*; reduced *mTOR*), and promoted cellular senescence. Importantly, *SELENOT* knockdown abolished the protective effects of Se under Pb stress, demonstrating that *SELENOT* is necessary for Se’s anti-aging action. These findings establish *SELENOT* as a bona fide suppressor of inflammation-driven and autophagy-mediated renal aging. Mechanistically, *SELENOT* likely acts at the intersection of redox sensing and membrane signaling, given its strategic subcellular localization. Its ability to modulate both inflammatory cytokines and autophagic flux suggests that *SELENOT* functions as a master integrator of stress responses in the kidney.

### 4.4. Integrating Oxidative Stress, HSPs, and the SELENOT-Centered Network

This work also revealed that Pb-induced oxidative stress was accompanied by elevated heat shock proteins (*HSP27*, *HSP40*, *HSP60*, *HSP70*, *HSP90*), which are known to respond to proteotoxic stress. The case of antioxidant decrease and oxidant increase is a reflection of the occurrence of oxidative stress in organisms. T-AOC is used to evaluate antioxidant capacity in organisms. GST and CAT are important antioxidant enzymes against oxidative stress. H_2_O_2_ is a typical oxidant. In the experiment, Pb treatment resulted in reduced T-AOC, GST, and CAT, and elevated H_2_O_2_, confirming that Pb induced oxidative stress in chicken kidneys. Consistent with these findings, Pb reduced CAT and caused oxidative stress in *C. argus* gills, livers, and kidneys [50]. Cadmium exposure led to decreased GST and CAT, increased H_2_O_2_, and oxidative stress in common carp hearts [46]. Interestingly, the decrease in the levels of GST and CAT may be due to the direct binding of Pb to their sulfhydryl groups [51], which alters their function and suppresses their activities [52]. Therefore, the deficit in antioxidant activity may be one of the direct consequences of Pb toxicity in chicken kidneys, and requires further investigation. In addition, in an oxidative stress model of *Labeo rohita* hepatocytes established by H_2_O_2_ treatment, the expressions of *HSP70* and *HSP90* were upregulated [53]. Treatment with *HSP70* decreased *IL-2* secretion and increased *IL-4* secretion in mouse ears [54]. These results also indicated that oxidative stress mediated *HSPs* elevation under Pb stress. Therefore, this study propose a hierarchical stress cascade: Pb-oxidative stress–HSP elevation–inflammatory dysregulation–autophagic overactivation–renal aging. Within this cascade, *SELENOT* appears to exert upstream or mid-stream control, as its loss amplifies all downstream events. Importantly, Se supplementation reversed *HSPs* elevation, further supporting that Se acts through selenoproteins to restore proteostasis and redox balance. The fact that *SELENOT* knockdown abrogated Se’s benefits places *SELENOT* as an important mediator in this pathway.

### 4.5. Limitations of the Study

Despite the novel findings that membrane-bound selenoprotein *SELENOT* plays a critical role in Se-mediated protection against Pb-induced renal aging, several limitations of this study should be acknowledged. First, only male chickens were used as the animal model. Although both male and female chickens at 90 days of age have not yet reached sexual maturity and endogenous sex hormone levels remain relatively stable, whether the conclusions can be directly extrapolated to females requires further investigation. Second, the Pb exposure dose (350 mg/L lead acetate in drinking water) and Se supplementation dose (1.00 mg/kg in diet) used in this study are relatively high, which were selected to establish a clear toxicological model and intervention effect. However, caution should be exercised when extrapolating these findings to environmental low-dose, long-term exposure scenarios. Third, the functional role of *SELENOT* was primarily verified using a knockdown strategy, which demonstrated that *SELENOT* is necessary for protecting against Pb-induced inflammation, autophagy, and cellular senescence. Nevertheless, gain-of-function experiments (e.g., overexpression of *SELENOT*) are still needed to further confirm the sufficiency of its protective effects. Fourth, the in vivo experiments were conducted exclusively in chickens, and the in vitro experiments were performed only in HK-2 human renal epithelial cells. Whether the mechanistic findings are conserved across different species, particularly in mammalian models (e.g., rodents) and other renal cell lines, remains to be established. Collectively, future studies employing female animals, environmentally relevant exposure doses, multiple animal models, and *SELENOT* overexpression strategies are warranted to systematically validate the core findings of this study and strengthen the translational potential of *SELENOT* as a therapeutic target for Pb-induced nephrotoxicity.

## 5. Conclusions

In summary, this study provides the first comprehensive evidence that membrane-bound selenoproteins, particularly *SELENOT*, are critical mediators of Se’s protection against Pb-induced renal aging. We demonstrate that Pb suppresses *SELENOT* expression, leading to unchecked inflammation and excessive autophagy, which converge to accelerate renal senescence. Se restores *SELENOT* expression, thereby rebalancing these processes. Our findings not only identify *SELENOT* as a sensitive and functional biomarker of Pb nephrotoxicity but also open new avenues for the therapeutic targeting of membrane-bound selenoproteins in heavy metal-induced kidney diseases. Future studies should explore the upstream transcriptional regulation of *SELENOT* under Pb stress and its potential interactions with other post-translational modifications in aging kidneys.

## Data Availability

The data are available from the corresponding author upon reasonable request.

## Supplementary Materials

The following supporting information can be downloaded at: https://www.mdpi.com/article/10.3390/biology15151218/s1. Table S1. The composition of standard commercial diet for chicken. Table S2. Primer sequences used for real-time PCR analysis of chicken. Table S3. Primer sequences used for real-time PCR analysis of human HK-2 cell line genes.
