# Supplementary material for: SELENOT, a Key Membrane-Bound Selenoprotein, Mediates Selenium’s Protection Against Lead-Induced Renal Aging in Chickens by Modulating Inflammation and Autophagy
Source: Biology (Basel). 2026 Jul 23;15(15):1218. doi: 10.3390/biology15151218 (PMC13463916; doi:10.3390/biology15151218)
Supplement: Supplementary file 1 [file biology-15-01218-s001.zip › biology-4434068-supplementary.pdf]

Table S1. The composition of standard commercial diet for chicken.

	1-28 days	29-97 days
crude protein (%)	20.5	19.3
moisture (%)	12.1	12.8
crude fiber (%)	4.3	5.5
crude ash (%)	6.8	7.8
calcium (%)	0.96	0.89
total phosphorous (%)	0.67	0.65
sodium chloride (%)	0.35	0.38
copper (mg/kg)	7.45	7.45
iron (mg/kg)	75.52	75.52
zinc (mg/kg)	45.15	45.15
manganese (mg/kg)	58.41	58.41
iodine (mg/kg)	0.37	0.37
selenium (mg/kg)	0.49	0.49
vitamin A (IU)	2750	2750
vitamin D3 (IU)	410	410
vitamin E (mg/kg)	12.51	12.51
vitamin K (mg/kg)	0.60	0.60
vitamin B1 (mg/kg)	1.73	1.73
vitamin B2 (mg/kg)	7.80	4.01
vitamin B12 (mg/kg)	0.01	0.01
nicotinic acid (mg/kg)	31.26	31.26
folic acid (mg/kg)	0.62	0.62
pantothenic acid (mg/kg)	11.50	11.50
biotin (mg/kg)	0.17	0.17

Table S2. Primer sequences used for real-time PCR analysis of chicken

Gene name	Accession number	Primer sequence
IL-2	AY510091	Forward 5'- GAACCTCAAGAGTCTTACGGGTCTA -3'<br>Reverse 5'- ACAAAGTTGGTCAGTTCATGGAGA -3'
IL-4	AJ621249	Forward 5'- GTGCCCACGCTGTGCTTAC -3'<br>Reverse 5'- AGGAAACCTCTCCCTGGATGTC -3'
IL-12β	AJ564202	Forward 5'- TGTCTCACCTGCTATTTGCCTTAC -3'<br>Reverse5'-CATACACATTCTCTCTAAGTTTCCACTGT -3'
HSP27	NM_205290.1	Forward 5'-ACACGAGGAGAAACAGGATGAG-3'<br>Reverse 5'-ACTGGATGGCTGGCTTGG -3'
HSP40	NM_001199325.1	Forward 5'- GGGCATTCAACAGCATAGA -3'<br>Reverse 5'- TTCACATCCCCAAGTTTAGG -3'
HSP60	NM_001012916.1	Forward 5' – AGCCAAAGGGCAGAAATG -3'<br>Reverse 5'- TACAGCAACAACCTGAAGA -3'
HSP70	NM_001006685.1	Forward 5'- CGGGCAAGTTTGACCTAA -3'<br>Reverse 5'- TTGGCTCCCACCCTATCTCT -3'
HSP90	NM_001109785.1	Forward 5'- TCCTGTCCTGGCTTTAGTTT -3'<br>Reverse 5'- AGGTGGCATCTCCTCGGT -3'
ATG5	gi|449273598	Forward 5'- GATGAAATAACTGAAAGGGAAGC -3'<br>Reverse 5'- TGAAGATCAAAGAGCAAACCAA-3'
Beclin 1	NM_001006332.1	Forward 5'- CAGACACGCTGCTGGACC -3'<br>Reverse 5'- TCTCCTTGTCATCCTCGTTCA -3'
Dynein	NM_001006519.1	Forward 5'- TGGGATAATCGCAGCAATAAGA -3'<br>Reverse 5'- AGGGAAGGACATGCAAGTAACAG -3'
LC3-II	NM_001031461.1	Forward 5 - CTTCTTCCTCCTGGTGAACG-3'<br>Reverse 5'- GCACTCCGAAAGTCTCCTGA-3'

mTOR	XM_417614.5	Forward 5'- GGACTCTTCCCTGCTGGCTAA -3'<br>Reverse 5'- TACGGGTGCCCTGGTTCTG -3'
GAPDH	NM_204305.1	Forward 5 - AGAACATCATCCCAGCGT -3'<br>Reverse 5'- AGCCTTCACTACCCTCTTG -3'

Table S3. Primer sequences used for real-time PCR analysis of human HK-2 cell line genes.

Gene name	Accession number	Primer sequence
TP53	NM_0012873 69.1	Forward 5'-CCATGGAGGAGCCGCAGTCAG -3'<br>Reverse 5'- GTGGATGATGGTGGTGCAGT -3'
CDKN1A	NM_204396.2	Forward 5'-GCGTGCAGGAACCTCTTCG -3'<br>Reverse 5'- CAGCTCCTGGCCTCAACAG -3'
CDKN2A	NM_000077.5	Forward 5'-ATGGAGCCTTCGGCTGACTG -3'<br>Reverse 5'- GTCACGGGTCGGGTGAGAG -3'
CDKN2B	NM_004936.4	Forward 5'-GCTACGCGCTTTTTCCCAG -3'<br>Reverse 5'- GTAGTTAGCGGCGCTCTGC -3'
H2AFX	NM_002105.3	Forward 5'-GGCGGCAAGGCCCGCG -3'<br>Reverse 5'-CTCCGCAGCCAGGATCTCG -3'
LMNB1	NM_005573.4	Forward 5'-GCAGGTGACGGAGCGCGAG -3'<br>Reverse 5'- GCAGCATCGTCCAGCGCG -3'
SELENOI	NM_033505.4	Forward 5'-GCCTGGATAGTTGGTCATGTG -3'<br>Reverse 5'- CAGGAAGGGTTTCATACCAGGC -3'
SELENOK	NM_021237.5	Forward 5'-GGTGACGTGAACGGCAGAAG -3'<br>Reverse 5'- AGAGGTGGCACCAGGATTGA -3'
SELENOS	NM_018445.6	Forward 5'-AGGAGGCGGAGCTTGCTCCT -3'<br>Reverse 5'- CCACCACCTCACCCCTTGG - 3'
SELENOT	NM_016275.5	Forward 5'-GCGGTCCGGAGCGAGG -3'<br>Reverse 5'- TGTCTATATATTGGTTGAGGGAGG -3'
DIO1	NM_000792.7	Forward 5'-AGGGTGCAGGTGGCATGTG -3'<br>Reverse 5'- GAAGGTCAGGTACAACTTCCAAA -3'
DIO3	NM_001362.4	Forward 5'-CGTGCTCTTCCCGCGCTT -3'<br>Reverse 5'- CTCGCCCTTCTCGGCCTTG -3'
IL-2	NM_000586.4	Forward 5'-CTACAAAGAAAACACAGCTACAACT-3'<br>Reverse 5'- TGTTTTCAGATCCCTTTAGTTCCAG-3'
IL-4	NM_000589.4	Forward 5'-ACCTTACAGGAGATCATCAAAACTTT-3'<br>Reverse 5'-CGGCAGCAAAGATGTCTGTTAC-3'
IL-12β	NM_002187.3	Forward 5'-TGGTCCACTGATATTTTAAAGGACC-3<br>Reverse 5'-TGTCAGCCACCAGCAGGTG-3'
ATG5	NM_004849.4	Forward 5'-GTTTGGACGAATTCCAACTTGTTTC-3'

		Reverse 5’-CATCTCGGATATGTCTTCTTGCC-3’
Beclin 1	NM_003766.5	Forward 5’-GAGGGATGGAAGGGTCTAAGAC-3’
		Reverse 5’-TGGTGACACGGTCCAGGAT-3’
Dynein	NM_0010374<br>94.2	Forward 5’-CGACCGCAGGCTGACCCC-3’
		Reverse 5’-GCGCAGCACAGCCTATGCTC-3’
LC3-II	NM_022818.5	Forward 5’-CCGTCGGAGAAGACCTTCAAG-3’
		Reverse 5’-GGCGTTAATGATCTTGATGAGCTC-3’
mTOR	NM_004958.5	Forward 5’-TGCAGGCTTCGAGTACCTGT-3’
		Reverse 5’-GGTTGTCTGTCACTTCCGGT-3’
GAPDH	NM_002046.7	Forward 5’-AGCCACATCGCTCAGACACC -3’
		Reverse 5’-GCCCAATACGACCAAATCCG -3’
